# A deeper insight into the sialome of male and female *Culex quinquefasciatus* mosquitoes

**DOI:** 10.1186/s12864-023-09236-1

**Published:** 2023-03-20

**Authors:** Stephen Lu, Ines Martin-Martin, Jose M. Ribeiro, Eric Calvo

**Affiliations:** 1 Laboratory of Malaria and Vector Research, National Institute of Allergy and Infectious Diseases, Bethesda, MD USA; 2 Laboratory of Medical Entomology, National Center for Microbiology, Instituto de Salud Carlos III, Madrid, Spain

**Keywords:** Hematophagy, Arthropod, Saliva, Endonuclease, Medical entomolgy, Evolution

## Abstract

**Introduction:**

During evolution, blood-feeding arthropods developed a complex salivary mixture that can interfere with host haemostatic and immune response, favoring blood acquisition and pathogen transmission. Therefore, a survey of the salivary gland contents can lead to the identification of molecules with potent pharmacological activity in addition to increase our understanding of the molecular mechanisms underlying the hematophagic behaviour of arthropods. The southern house mosquito, *Culex quinquefasciatus*, is a vector of several pathogenic agents, including viruses and filarial parasites that can affect humans and wild animals.

**Results:**

Previously, a Sanger-based transcriptome of the salivary glands (sialome) of adult C. quinquefasciatus females was published based on the sequencing of 503 clones organized into 281 clusters. Here, we revisited the southern mosquito sialome using an Illumina-based RNA-sequencing approach of both male and female salivary glands. Our analysis resulted in the identification of 7,539 coding DNA sequences (CDS) that were functionally annotated into 25 classes, in addition to 159 long non-coding RNA (LncRNA). Additionally, comparison of male and female libraries allowed the identification of female-enriched transcripts that are potentially related to blood acquisition and/or pathogen transmission.

**Conclusion:**

Together, these findings represent an extended reference for the identification and characterization of the proteins containing relevant pharmacological activity in the salivary glands of *C. quinquefasciatus* mosquitoes.

**Supplementary Information:**

The online version contains supplementary material available at 10.1186/s12864-023-09236-1.

## Introduction

Blood acquisition is a pharmacological endeavor for any hematophagous arthropod. Upon piercing the host’s skin, several defensive mechanisms are deployed, including vasoconstriction, platelet activation and blood coagulation leading to a reduction of the overall blood flow at the bite site. Additionally, a complex signaling pathway also stimulates inflammatory and immune responses that can be detrimental to the blood feeding arthropod. Over the last decades, the structural and biochemical study of salivary proteins established that hematophagous vectors convergently evolved a complex and distinct salivary mixture that can interfere with host hemostatic and immune responses, leading to the postulation that all blood feeders have at least an anticoagulant, an inhibitor of platelet aggregation and a vasodilator in their saliva [1]. In addition to their role in blood acquisition, salivary proteins from mosquitoes have been shown to facilitate pathogen transmission to vertebrate hosts [2–5] and, therefore, can be considered potential targets for vaccine development.

The mosquito *Culex quinquefasciatus*, commonly referred as the southern house mosquito, is a vector of several pathogenic agents that affect both humans and wild animals including the West Nile virus, St. Louis encephalitis virus, *Wuchereria bancrofti* and *Plasmodium relictum* [6–8]. However, the salivary activity of *C. quinquefasciatus* has been less explored than other medical relevant mosquitoes such as *Anopheles* sp. and *Aedes* sp., with only a few proteins characterized [9–12]. Although the southern mosquito genome is available [13], there is a limited knowledge regarding the sequences of genes expressed in the salivary gland with only a Sanger-based sialome of *C. quinquefasciatus* females being available [14]. Therefore, we present here a more comprehensive transcriptome analysis of both male and female adult mosquitoes based on an Illumina RNA-sequence approach. We carried out a *de novo* assembly of over 100 million high quality reads and combined the extracted CDS with the ones derived from the genome assembly, resulting in an extensive reference of *C. quinquefasciatus* salivary gland contents. A total of 7,539 CDS were identified and made publicly available. Finally, the comparison between male and female transcripts allowed the identification of sex-specific transcripts that might aid in the identification and characterization of pharmacologically active salivary proteins, proteins that can affect pathogen transmission as well as determine potential biomarkers of mosquito exposure. Additionally, comparison of mosquito sialomes could also provide insights regarding the evolution of the hematophagic behaviour within the Diptera order.

## Materials and methods

### Mosquitoes rearing and salivary glands collection

A *C. quinquefasciatus* colony was established in 2015 from egg rafts collected in Hilo, Hawaii, US and maintained at the Laboratory of Malaria and Vector Research insectary, NIAID, NIH. The mosquitoes were reared using 10% Karo Syrup prior to dissection. The salivary glands were dissected from 1- to 3-day old mosquitoes (between 8 and 10 AM) in sterile PBS pH 7.4 and immediately transferred to an Eppendorf tube containing 100 µl of TRIzol (Invitrogen). Pools of 50 pairs of female salivary glands and 100 pairs of male salivary glands were stored at − 80 °C until RNA extraction. More male than female salivary glands were used to account for their overall lower total RNA amount [15, 16]. Three biological replicates of male and female samples were collected.

### Library preparation, sequencing, and analysis

Total RNA from mosquito salivary glands was isolated using TRIzol (Invitrogen) according to the manufacturer instructions. RNA purity was assessed with the NanoPhotometer® spectrophotometer (IMPLEN, CA, US), and RNA integrity and quantification were assessed using the RNA Nano 6000 Assay Kit of the Bioanalyzer 2100 system (Agilent Technologies, CA, US). Sequencing libraries were generated using NEBNext® UltraTMRNALibrary Prep Kit for Illumina® (NEB, US) following manufacturer’s instructions. Briefly, mRNA was purified from total RNA using poly-T oligo-attached magnetic beads. Fragmentation was carried out using divalent cations under elevated temperature in NEBNext First Strand Synthesis Reaction Buffer (5X) or by sonication with Diagenode Bioruptor Pico for breaking RNA strands. First strand cDNA was synthesized using random hexamer primer and M-MuLV Reverse Transcriptase (RNase H-). Second strand cDNA synthesis was subsequently performed using DNA Polymerase I and RNase H. Remaining overhangs were converted into blunt ends via exonuclease/polymerase activities. After adenylation of 3’ ends of DNA fragments, NEB Next Adaptor with hairpin loop structure were ligated to prepare for hybridization. To select cDNA fragments of preferentially 150 ~ 200 bp in length, the library fragments were purified with AMPure XP system (Beckman Coulter, Beverly, US). Three microliters of USER Enzyme (NEB, US) were used with size-selected, adaptor-ligated cDNA at 37 °C for 15 min followed by 5 min at 95 °C before PCR. PCR was performed with Phusion High-Fidelity DNA polymerase, Universal PCR primers and Index (X) Primer. PCR products were purified (AMPure XP system) and library quality was assessed on the Agilent Bioanalyzer 2100 system. The Illumina reads were trimmed of the Illumina adapters and any low-quality sequences (Q < 20) using TrimGalore (https://github.com/FelixKrueger/TrimGalore), merged into a single file and assembled using ABySS (V2.3.1) [17] with k values from 25 to 95 (with increments of 10) in single-stranded mode and Trinity (V2.9.0) [18] in single-stranded F mode. The assemblies from ABySS and Trinity were combined and filtered using CD-HIT [19]. Coding DNA sequences (CDS) with an open reading frame (ORF) of at least 150 nucleotides were extracted based on BLASTp results to several databases, including a subset of the non-redundant protein database, the transcriptome shotgun assembly (TSA) and the Refseq-invertebrate. CDS were extracted if sequences presented at least 70% of coverage with a matching protein. Additionally, all ORFs starting with a methionine and with 40 amino acids in length were submitted to the signalP tool (V3.0). Sequences that presented a putative signal peptide were mapped to the ORFs and the most 5’ methionine was selected as the starting of the transcript [20]. The extracted CDS from our *de novo* assembly were merged with the ones annotated in the genome (assembly Cqui1.0, Genbank: GCA_015732745.1) and the redundant sequences were filtered using the CD-HIT tool (considering 95% of identity). For annotation we used an in-house program that scans a vocabulary of ~ 400 words and their order of appearance in the protein matches from BLAST results against several databases (TSA, subset of the NR, Refseq-protozoa, Refseq-invertebrate, Refseq-vertebrate, uniport, MEROPS, PFAM, Smart and CDD), including their e-values and coverage. Relative quantification of CDS was performed by mapping the trimmed library reads to the extracted CDS using the RSEM tool [21]. The annotated CDSs were exported to a hyperlinked Excel spreadsheet that is currently available for download (Supplementary file 1).

### Tridimensional structure prediction

Molecular modeling was performed using AlphaFold2 [22]. The superposition of modeled proteins with protein structures deposited on the Protein Data Bank (PDB) were prepared using PyMol (2.4).

### Statistical analysis

Differentially expressed transcripts analysis between females and males was carried out using the edgeR package [23] for R [24]. Briefly, we generate a counting matrix of each CDS by mapping the trimmed Illumina reads to the final extracted CDS using RSEM. We them extracted the count data of CDS that presented an average TPM ≥ 3 in male or female samples and used this filtered matrix as input for edgeR. Finally, the cout data of each filtered CDS was normalized by the library size respective library using the edgeR function calcNormFactors(). For the identification of differently expressed transcripts we used the generalized linear model approach of edgeR with dispersion estimated by the Cox-Reid profile-adjusted likelihood. Transcripts were considered differentially expressed when presented a LogFC ≥ ± 2, the p-value and the false discovery rate (FDR) were less than 0.05. The phylogenetic tree was constructed using the maximum likelihood model [25] with MegaX [26]. The amino acid alignments were performed with Clustal omega [27] and edited with Bioedit [28].

## Results and discussion

### Overall description of ***C. quinquefasciatus*** sialome

Illumina sequencing of six libraries from *C. quinquefasciatus* salivary glands resulted in 105,064,955 high quality reads. Our *de novo* assembly using ABySS and Trinity generated 34,074 sequences from which a total of 21,106 potential CDS were extracted. After merging our *de novo* CDS with those derived from the genome assembly (assembly Cqui1.0, Genbank: GCA_015732745.1) we obtained 31,426 putative CDS and 2,100 sequences annotated in the genome as non-coding RNA (ncRNA). The quantification of each transcript was estimated using the Transcript Per Million (TPM) measurement by mapping the trimmed Illumina reads to the final list of transcripts using the RSEM tool. The levels mapped reads were similar between our male and female samples (64.5% ± 0.6),with exception of one female sample (F1), which presented only 49.4% of mapped reads, indicating a possible bias with this sample. Despite this discrepancy, the values of mapped reads observed here are within range of other sialome from blood feeding arthropods [16, 29] and indicate that no major bias is present in our pipeline. It is noteworthy that the remaining unmapped reads could be potentially from 5` and 3` UTRs or putative CDS that we failed to identify due to lack of homology to previous deposited sequences, lack of a putative signal peptide or having an ORF with less than 150 nucleotides. Initial exploration of the data using multi dimension analysis and the heatmap plot based on the relative quantification of each transcript confirmed that sample F1 was not a suitable biological replicate (Supplementary Fig. 1) and, therefore, it was excluded from the differentially expression analysis.

The putative CDS that had an average TPM of at least 3 in males or female samples were extracted, resulting in 7,539 CDS that were functionally classified into 25 groups and 159 ncRNA (Supplementary file 1). As observed in the Sanger-based *C. quinquefasciatus* and in the sialome of other blood feeding vectors [14, 15, 30, 31], the “secreted” functional class was the major component of the mosquito salivary glands homogenates (Table 1). In females this class represented 61.2% of all quantified CDS, while in males it accounted for 56%. The second most abundant classes were the “unknown” class (4.63%) and the “protein synthesis” class (8.77%) in females and males, respectively. Finally, the third most abundant class in females (4.55%) was the “protein synthesis”, while in male was the “unknown” class (5.59%). It’s noteworthy that sequences classified within the “unknown” group are of particular interest, since they presented low or no identity to previously deposited sequences and therefore represent potential novel mosquito sequences.

**Table 1:** Functional classification of transcripts found in the salivary glands of *C. quinquefasciatus* mosquitoes

	Females		Males		
Class	Average No. Transcripts	Average TPM (%)	Average No. Transcripts	Average TPM (%)	No. of DEG
Cytoskeletal	198.5	0.64	199.3	0.69	9
Extracellular matrix/cell adhesion	110	0.57	106.7	0.64	21
Immunity	144	1.46	143.0	2.64	16
Metabolism, amino acid	150.5	0.87	149.0	0.96	14
Metabolism, carbohydrate	150	0.99	149.6	1.28	21
Metabolism, energy	416.5	2.77	416.3	3.32	6
Metabolism, intermediate	89	0.23	88.7	0.21	4
Metabolism, lipid	273	1.31	266.3	1.10	26
Metabolism, nucleotide	91	3.12	88.7	1.01	5
LncRNA	156.5	2.63	156.0	1.65	17
Nuclear export	18	0.02	18.0	0.02	-
Nuclear regulation	167.5	0.51	168.7	1.26	3
Oxidant metabolism/detoxification	137.5	0.65	134.0	0.57	16
Proteasome machinery	304	1.47	302.3	1.51	5
Protein export machinery	424	2.65	418.7	2.17	11
Protein modification machinery	217	2.32	214.3	1.90	10
Protein synthesis machinery	377.5	4.55	375.3	8.77	4
Secreted	847.5	61.21	817.7	56.00	173
Signal transduction	548.5	1.77	543.0	2.04	50
Storage	17	1.89	17.0	0.32	2
Transcription factor	31	0.04	30.7	0.04	1
Transcription machinery	752.5	1.60	753.7	2.20	12
Transporters	290	1.96	281.7	2.36	33
Transposable element	30	0.09	30.7	0.02	6
Unknown	1536.5	4.63	1521.7	5.59	101
Unknown, conserved	27	0.04	27.0	0.05	1

Further inspection of the “secreted” class revealed the presence of several protein families commonly reported in the sialome of other mosquitoes, including the Antigen-5, the 30 kDa salivary allergen, D7, amylases, maltases, apyrases, peptidases, and peptidases inhibitors (Table 2) [14–16, 32]. In females, the most abundant secreted protein families were the ones implicated in blood acquisition, such as the D7, with 13 CDS accounting for 26.6% of all secreted CDS, and the aegyptin-like/30 kDa salivary allergen (3 CDS representing 18%). In males the “unknown” and “unknown, conserved” classes accounted for ~ 53.9% of all secreted proteins identified, highlighting the major gap in the knowledge of males’ salivary glands contents.

**Table 2:** Families of putative secreted proteins within the sialotranscriptome of *C. quinquefasciatus*

	Female		Male				
Family	No. CDS	Avg.TPM	No. CDS	Avg.TPM	No. of DEG	Female TPM / (1 + Male TPM)	Male TPM
							/ (1 + Female TPM)
7.7 kDa	1	1,506.50	1	154.8	1	9.67	0.1
7.8 kDa	3	2,125.20	3	0.3	3	1,585.97	0
9.6 kDa	1	3,202.90	1	0.9	1	1,694.66	0
9.7 kDa	1	484.3	2	0.2	1	413.93	0
13.1 kDa	1	68.7	-	0	1	68.7	0
15.3 kDa	4	34,705.80	2	2.8	4	9,109.13	0
16 kDa	3	74	2	20.3	1	3.47	0.27
16.8 kDa	1	26	1	3.1	-	6.28	0.12
17.5 kDa	1	1,022.30	1	0.1	1	896.75	0
20.2 kDa	1	3,664.50	1	141	1	25.81	0.04
23.4 kDa	1	527.6	1	1,142.40	-	0.46	2.16
27 kDa	4	333.4	4	385.4	-	0.86	1.15
30 kDa	3	97,499.30	3	431.7	3	225.33	0
30.5 kDa	2	2,719.90	2	8,791.10	-	0.31	3.23
34 kDa	1	1,076.10	1	8.2	1	116.97	0.01
41 kDa	1	3,398.50	1	2,898.20	-	1.17	0.85
56 kDa	1	7,906.30	1	13,738.00	-	0.58	1.74
62 kDa	1	837.1			1	837.1	0
Antigen-5	7	39,914.50	7	12,271.70	7	3.25	0.31
Basic-tail	1	942.3	1	30.1	1	30.27	0.03
D7	13	144,368.0	13	281.1	11	511.76	0
gSG1	1	101.7	1	0.1	1	96.86	0
gSG5	2	3,161.20	2	3.5	2	702.49	0
Hormone binding	9	354.8	9	448.9	2	0.79	1.26
Immunity	1	5,062.70	1	14,028.50	-	0.36	2.77
Enzymes							
Acid phosphatase	1	119.5	1	31.5	1	3.68	0.26
Apyrase	4	231.1	4	43.3	2	5.22	0.19
ACE	1	1.5	1	8.6	-	0.15	3.47
Cysteine peptidase	12	2,100.80	15	2,246.70	-	0.93	1.07
Dipeptidyl peptidase	1	95.1	1	53.5	-	1.74	0.56
Prolyl endopeptidase	1	18.8	1	11.1	-	1.55	0.56
Furin	2	12.4	2	13.4	-	0.86	1
Metalloprotease	5	91.3	4	47	1	1.9	0.51
Serine peptidase	124	5,838.40	124	9,498.70	11	0.61	1.63
Other proteases	27	459.6	27	827.1	3	0.56	1.8
Phospholipase	10	538.2	10	536.1	3	1	0.99
Esterases/Lipases	13	4,691.60	13	16,007.60	3	0.29	3.41
Sphingomyelinase	5	2,094.10	5	661.9	-	3.16	0.32
Amylase	6	13466.4	6	53882.4	1	0.25	4
Maltase	4	30051.7	4	97,402	-	0.313	3.24
Protease Inhibitor						0	0
Cystatin	1	32.4	3	81.2	-	0.39	2.43
Kazal	4	320.1	4	1,027.00	-	0.31	3.2
Pacifastin	1	165	1	12.8	1	11.96	0.08
Serpin	19	15,256.00	19	579.1	4	26.3	0.04
TIL	2	8,561.50	2	21,387.40	-	0.4	2.5
TIMP	0	0	1	4.5	-	0	4.53
Leu-rich	10	317.4	10	112	3	2.81	0.35
Mucin	16	21,894.40	16	76,449.60	4	0.29	3.49
OBP	18	481.4	18	4,789.40	8	0.1	9.93
Pro-rich	2	3,377.50	2	55.9	1	59.36	0.02
Scoloptoxin	4	343.3	4	353.9	-	0.97	1.03
SGS3	1	4.7	1	0.4	-	3.38	0.07
Unknown, conserved	227	56,737.20	228	71,011.20	48	0.8	1.25
Unknown	211	38,304.40	216		22	0.25	3.92
WRP	12	24,892.40	6	7.2	12	3,031.96	0

Comparison of the relative CDS quantification between males and females using the edgeR package led to the identification of 559 differentially expressed transcripts (LogFC ≥ ± 2, p-value < 0.05 and FDR < 0.05, Supplementary Fig. 1C), from which 398 were up-regulated and 161 down-regulated in females (Supplementary file 2). Additionally, we also identified 17 ncRNA differentially expressed between the two groups (12 up-regulated and 5 down-regulated in females). The functional classification of the differentially expressed CDS is summarized in Table 1, in which the “secreted” class showed the highest number of differentially expressed transcripts (173 CDS) between the two groups. Considering that only female mosquitoes seek blood, the identification of unique or highly abundant CDS in their salivary glands could lead to the identification of molecules containing potent pharmacology activity relevant for blood acquisition and/or related to pathogen transmission. Therefore, in the next section, we present a discussion of the putative secreted salivary protein families that were differentially expressed in female and male mosquitoes.

### Secreted transcripts differentially expressed in the salivary glands of ***C. quinquefasciatus*** females and males

#### D7 protein family

The D7 protein family is related to the odorant-binding proteins (OBP) superfamily [33], and was identified for the first time in the salivary glands of *Aedes aegypti* [34]. Members of this protein family are subclassified based on the number of OBP-like domains they possess; short-forms contain a single domain and often present 15–20 kDa, while the long-forms D7 have two OBP-like domains and range from 24 to 30 kDa. The D7 protein family is commonly found in the salivary gland of mosquitoes where they are among the most abundant protein families [15, 16, 32, 35]. In anopheline mosquitoes the short forms are predominant, while in culicines, transcripts coding for long-D7 are the most abundant ones. The first *C. quinquefasciatus* sialome [14] reported 11 CDS belonging to this family, from which four were subclassified as short-form D7 and seven as long-forms. In the current data set we identified a total of 13 CDS coding for D7-like proteins (8 long, 5 short, Supplementary Table 1), and, as observed in other mosquitoes, most of the D7 proteins were up-regulated in females’ salivary glands (Table 2), reinforcing their role in blood acquisition.

Over the last decade several members of the D7 protein family have been functionally and structurally characterized and shown to act as *kratagonists*, proteins capable of binding and trapping small agonists relevant for host homeostasis such as biogenic amines and leukotrienes [36–40]. Recently, two long-D7 from *C. quinquefasciatus* were described as kratagonists [10]. The CxD7L1 (reported here as XP_038114211.1) was shown to specifically bind to adenine, adenosine, AMP, ADP, and ATP, while CxD7L2 (reported here as XP_001865413.2) was shown to interact with biogenic amines (serotonin, histamine, and epinephrine) in addition to cysteinyl leukotrienes (LTC_4_, LTD_4_ and LTE_4_). Currently, CxD7L1 is the only D7 protein shown to bind to ADP and ATP [10]. Considering the low levels of apyrases observed *C. quinquefasciatus* salivary glands when compared to other mosquitoes, it is possible that they selected a kratagonist as a platelet aggregation inhibitor, as they would be more efficient in removing low concentrations of ADP (1 µM) than apyrases [41].

Putative short-D7 proteins were also reported to be up-regulated in the salivary glands of females culicine mosquitoes when compared to males [14, 15]. Here we found three short-forms highly expressed in females’ salivary glands (LogFC 12.9–13.6, Supplementary file 2), suggesting a potential role in blood acquisition. It’s noteworthy that recombinant short-D7 from *Ae. aegypti* and *Ae. albopictus* did not presented the biogenic binding activity found in the long-D7 of culicines [42]. Currently, no short-D7 has been functionally characterized in culicine mosquitoes and their role in mosquito physiology remains elusive. However, it was previously speculated that culicines short-D7 may not interact with biogenic amines since they lack the residues important for such interaction [43].

In addition to their sequestering activity, the role of D7 proteins during viral infection has also been explored. An initial transcriptome comparing the salivary glands of WNV-infected and non-infected *C. quinquefasciatus* females identified a long-D7 down-regulated 14- and 21-days post-infection [44]. In a second study, mice vaccinated with a recombinant long-D7 protein from *C. tarsalis* showed enhanced pathogenesis and higher mortality rates when compared to the control groups when exposed to WNV-infected mosquitoes. Additionally, passive immunization of mice with sera from D7-vaccinated mice also resulted in increased pathogenesis, indicating that such phenotype is related to antibodies targeting the D7 proteins [45]. The authors speculated that blocking the sequestering activity of D7 proteins would result in increased probing time, and, therefore, more viral particles are injected into the host. Thus, it appears that D7 proteins are not suitable targets for a pathogen-blocking vaccine.

Mapping of the D7 CDS to the genome assembly indicated that most D7 sequences are clustered in the chromosome 2 (CM024711.1, GCA_015732765.1), with exception of three CDS (XP_038120412.1, Cq-contig_13280 and XP_038111256.1) which are mapped in chromosome 3 (Supplementary file 1). Interesting, XP_038120412.1 was found to be similar to the juvenile hormone binding D7 from *Ae. aegypti* (Supplementary Fig. 2) [46] and presented an overall low expression in the salivary glands of both males (TPM = 27.54) and females (TPM = 70.82). Taken together, it’s possible the XP_038120412.1 is also acting as a juvenile hormone binding protein.

#### 30 kDa salivary allergen

Members of this protein family are usually among the most abundant CDS in mosquitoes sialomes [47, 48]. In the present study, they were the second most abundant salivary protein family. The previous *C. quinquefasciatus* sialome reported two CDS belonging to this protein family [14], while three CDS were identified in the current dataset, including the most abundant secreted transcript (XM_001845231) in the female salivary gland with an average TPM of 88,429 (Supplementary file 1). Together, these three CDS accounted for almost 18% of all secreted CDS identified in females (Table 2) and all three were found up-regulated in females (LogFC 3.3–10.8) (Supplementary file 2).

The function of the 30 kDa salivary allergen was initially described in other mosquitoes, *Ae. aegypti* (aegyptin) [49, 50] and *Anopheles stephensi* (AAPP) [51], as molecules capable of interacting with collagen at different binding sites, including the Von Willebrand factor, the glycoprotein VI and the integrin α2β1 [52] binding domains, inhibiting platelet aggregation. Members of this protein family were later characterized in other blood feeding Diptera such as the black fly, *Simulium nigrimanum*. Simplagrin was shown to bind to the collagen von Willebrand factor binding site, however, it failed to block GPVI and integrin α2β1 binding to collagen [53]. Similarly, the *Ae. albopictus* aegyptin-like protein named AlALP was shown to have an anticoagulant activity by prolonging the activate partial thromboplastin time (APTT), prothrombin time (PT) and thrombin time (TT), however its molecular mechanism remains unknown [54].

**Fig. 1:**
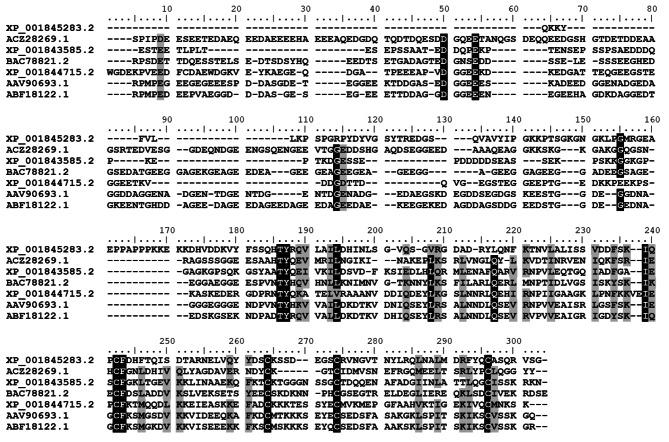
Amino acid alignment of members of the 30 kDa salivary allergen protein family from *C. quinquefasciatus* (XP_001845283.2, XP_001843585.2, XP_001844715.2), *Simulium nigrimanum* (ACZ28269.1), *Ae. albopictus* (AAV90693.1), *Ae. aegypti* (ABF18122.1) and *An. stephensi* (BAC78821.2). Identical and similar residues are highlighted

When comparing *C. quinquefasciatus* sequences to those from other Diptera vectors, we found that the predicted mature sequences have shorter N-termini, while some degree of similarity was found in the C-terminal region (Fig. 1). Considering that the C-terminal region is responsible for its collagen binding activity [55], it is likely that *C. quinquefasciatus* aegyptin-like proteins have a similar activity. It is important to note, however, that collagen binding activity has not been demonstrated in *C. quinquefasciatus* saliva or salivary glands homogenates.

Interestingly, although classified within this family, XP_001845283.2displays distinct features when compared to other 30 kDa salivary allergens. The predicted mature sequence has a theoretical isoelectric point of 8.94, while other aegyptin-like proteins have rich acidic N-termini conferring them a pI near 4. Tridimensional structure prediction using AlphaFold revealed that the C-terminal is arranged as four packed α-helices, which is also found in the salivary complement inhibitors of anopheline mosquitoes [56] (Supplementary Fig. 3), while the rest of the protein appears to be intrinsically disordered. In addition to the α-helices, XM_001845231 model presents three anti-parallel β-sheets that are not found in other members of this family (Supplementary Fig. 4). Finally, phylogenetic analysis of the 30 kDa salivary allergens positioned the XP_001845283.2 near simplagrin in a distinct clade from the *Ae. aegypti* and *Ae. albopictus* sequences (Supplementary Fig. 5), suggesting a distant evolutionary relationship between mosquito sequences.

Further biochemical studies are currently underway to uncover its function.

Similar to other salivary proteins, the possible involvement of the 30 kDa salivary allergen during viral transmission has been recently explored. A proteome comparison of DENV-2 infected and non-infected *Ae. aegypti* revealed that aegyptin is 3.5-fold less expressed in the salivary glands of infected mosquitoes [57]. Additionally, co-inoculation of recombinant aegyptin and DENV-2 in mice resulted in a reduction of DENV-2 particles at 2- and 3-days post-infection [58]. Although their exact mechanism is still unknown, these studies highlight their importance during viral transmission.

#### β-trefoil Salivary proteins

Proteins classified within this family had a predicted structure similar to the β-trefoil domain found in ricin (Supplementary Fig. 6). It is important to note that the CDS unified in this section were originally classified into different proteins families such as the tryptophan-rich proteins (WRP), 13.1 kDa, 15.3 kDa, 16 kDa and the 17 kDa protein families. Together, they represented the third most abundant secreted salivary protein group in females.

Among the protein families contained in this section, the 15.3 kDa and WRP protein families were the 6th and 7th most abundant secreted salivary protein families in females, accounting for 6.4% and 4.6%, respectively (Table 2). It is noteworthy that the CDS XM_038255158.1, classified here as a member of the 15.3 kDa family, was the fourth most abundant CDS in female salivary glands with an average TPM of 32,096 (Supplementary file 1), while only a small fraction was observed in male mosquitoes (average TPM = 2.7), suggesting a potential role in blood feeding.

The WRP protein family was reported for the first time in the previous *C. quinquefasciatus* sialome as a novel protein family with no identity to any other known eukaryotic sequence. However, PSI-BLAST analysis indicated their similarities to the bacterial proteins containing the trefoil domain [14]. The later sequencing and annotation of *C. quinquefasciatus* genome revealed the presence of 28 genes belonging to this family [13], and additional sequences over-expressed in female salivary glands were also reported within the recent *C. tarsalis* sialome [30]. Currently, BLASTp of theses CDS against the Diptera taxonomy group fails to return significant matches from *Aedes* and *Anopheles* mosquitoes, although similar sequence can be found in the frog biting fly *Corethrella appendiculata* [59] and in the mosquito *Psorophora albipes* [60]. Functionally, only two members of the WRP proteins have been characterized. The CqDVP-2 and CqDVP-4 transcripts were overexpressed in the salivary glands of *C. quinquefasciatus* females. Additional Western blot and immunofluorescence assays confirmed the presence of the native protein in the distal part of the lateral lobes of the salivary gland tissue, while mass spectrometry analysis of the mosquito saliva identified unique peptides from both proteins, confirming that they are indeed secreted into the host. The three-dimensional structure of both proteins was also solved by X-ray crystallography, confirming the presence of the β-trefoil fold and an initial functional screen suggests that they can bind to carbohydrates [11], however their biological function in blood feeding remains to be elucidated.

#### Antigen-5

Antigen-5-like proteins belong to the larger family of cysteine-rich extracellular proteins (CRISP) that are ubiquitously found in animals and plants [61] and are usually the main component of vespid venoms [62, 63]. In the previous *C. quinquefasciatus* sialome, three CDS classified as antigen-5-like proteins were identified [14]. Moreover, Edman degradation confirmed the presence of at least one protein in the salivary gland homogenates, suggesting that it is potentially secreted into the saliva and, consequently, into the host. In our current dataset we observed seven CDS belonging to this family and together they represented the fourth most abundant protein family in female salivary glands (TPM = 7.36%) and the 7th most abundant family in males (TPM = ~ 3%). The differential expression gene (DEG) analysis revealed that all seven CDS are differentially expressed between the two groups, with two (XM_001841639.2 and XM_001868651.2) transcripts up-regulated and the other five down-regulated in females.

Functionally, the role of salivary antigen-5-like proteins in mosquitoes remains elusive. Their presence in both male and female salivary glands and in the salivary glands of non-blood-feeding mosquitoes indicates they are not uniquely related to blood acquisition [14, 15, 30, 64]. However, evidence that the antigen-5-like proteins can contribute to blood acquisition has been demonstrated elsewhere. In the horse fly, *Tabanos yao*, salivary antigen-5 proteins interfered with platelet aggregation and thrombus formation [65, 66]; however, this was achieved by the acquisition of a disintegrin RGD domain. In *Dipetalogaster maxima* and *Triatoma infestans*, an antigen-5-like protein also inhibited platelet aggregation [67] and possessed a superoxide dismutase activity. Recently, an antigen-5-like salivary protein from *Ae. aegypti* was shown to facilitate Zika virus transmission in a mice model by inducing autophagy [68]. A second study demonstrated that the same protein can bind to the Zika virus envelope protein with high affinity [69], Suggesting that salivary antigen-5 proteins might favor viral transmission by different molecular mechanisms.

#### Serine peptidase inhibitors

Different classes of serine peptidase inhibitors are commonly described in the sialome of mosquitoes with a high range of TPM values [15, 16, 30]. Functionally, such inhibitors have been associated with the disruption of several biological processes such as host complement activation and blood clotting cascade [1]. Studies with salivary glands extracts of culicine mosquitoes demonstrate the presence of a specific factor Xa inhibitor that was later isolated and identified as a serpin [70–72]. In the previous *C. quinquefasciatus* sialome two CDS classified as serpins were reported [14], while in the current dataset we identified 19 sequences coding for putative serpins in both male and female mosquitoes (Table 2). Our DEG analysis revealed that four sequences were differentially expressed, from which three were up-regulated (LogFC 4.7–8.4) and one (Cq-contig_10400) was down-regulated in females (LogFC = -4.1). The latter should be carefully interpreted since the average TPM values of Cq-contig_10400 in male salivary glands were somewhat low (15.2 ± 6.26, Supplementary file 2), and, therefore, the protein might not be present in relevant physiological concentrations.

Interestingly, when comparing the overall expression of serpins in the salivary glands of female mosquitoes, we found that *C. quinquefasciatus* have a higher average TPM values than other culicine mosquitoes such as *Ae. aegypti*, *Ochlerotatus triseriatus* and *C. tarsalis* [15, 16, 30]. Although biochemical characterization is currently unavailable for *C. quinquefasciatus* serpin’s, it is very likely that at least one of them acts as an anti-clotting molecule by inhibiting factor Xa activity. Considering that serpins often display high specificity for their targets, it is possible that other serine peptidases are disrupted by the mosquito salivary serpins.

#### Apyrases

Apyrases are enzymes that target ATP and ADP catalyzing their degradation to AMP plus orthophosphate [73]. Members of this protein family are currently subclassified into three types: the *Cimex*-type apyrases, the homologs to the human B cell antigen CD-39 and the 5’-nucleotidase family [74]. In mosquitoes, such enzymes belong to the 5’-nucleotidase subfamily [1]. Functionally, apyrases have been shown to inhibit ADP-induced platelet aggregation, thus favoring blood acquisition [75]. Moreover, phylogenetic analysis of apyrases from different blood-feeding arthropods indicates that members of this protein family were convergently selected, highlighting their relevance for the hematophagic behavior [74].

In the current dataset we found three full-length CDS coding for putative apyrases/5’nucleotidases and one truncated CDS in the salivary gland of both males and females *C. quinquefasciatus* (Table 2), while only a single sequence was found in the previous sialome study [14]. The DEG analysis revealed that two of the four CDS were up-regulated in female mosquitoes (LogFC 2.57 and 5.53). Survey of salivary glands activity from different mosquitoes’ species (*C. quinquefasciatus*, *Ae. aegypti* and *An. albimanus*) revealed that *C. quinquefasciatus* had the least abundant ADPase activity among them [76]. In line with this observation, we found that the overall expression levels of apyrases from *C. quinquefasciatus* (TPM = 231.1) were drastically lower when compared to other female mosquitoes such *Ae. aegypti* (TPM = 1,049)[15] and *O. triseriatus* (TPM = 6,191)[16], which could explain why only one member of this family was found in the Sanger-based sialome.

Low levels of apyrases were also reported in the recent *C. tarsalis* sialome (RPKM = 184.44)[30], suggesting that apyrases activity is also reduced. Considering the preference of *Culex* mosquitoes for avian hosts [77] and that avian thrombocytes (analogs to the mammalian platelets) do not aggregate upon ADP or ATP exposure [78], it is possible that the apyrase genes in culicids are in a path to become a pseudo-gene [30].

#### Unknown and unknown conserved

**Fig. 2:**
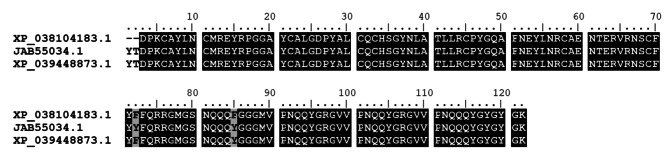
Amino acid alignment of unknown proteins from *C. quinquefasciatus (*XP_038104183.1), *C. pipiens pallens* (XP_039448873.1) and *C. appendiculata* (XP_039448873.1). Identical and similar residues are boxed

Members of this “functional class” represent CDS that we failed to classify in known protein families. The *unknown* class accounts for CDS that had low or no sequence identity to any previously deposited sequence and, therefore, can be considered potential novel sequences. While the *unknown conserved* class comprises sequences that presented high similarities to sequences of no known function reported elsewhere. In the current dataset both classes combined represented, approximately, 17.5% of all secreted CDS in females and 53.8% in males (Table 2), highlighting the significant knowledge gap between male and female salivary gland composition and function. DEG analysis revealed that 39 CDS were up-regulated in female salivary glands (Supplementary file 2). Among those sequences XP_038104183.1 was the 9th most abundant transcript coding for a putative salivary protein in female with an average TPM of 10,316.4 (Supplementary file 1). XP_038104183.1 was found to be almost identical to sequences from the frog biting fly *C. appendiculata* [59] and from *C. pipiens pallens* (Fig. 2). Currently, no function has been attributed to these proteins. However, their abundance in female salivary glands suggests a potential role in blood acquisition and/or pathogen transmission.

It is important to note that since potential novel sequences classified under the *unknown* class were obtained by our *de novo* assembly strategy, we cannot fully reject the possibility that they are artefacts or *chimeric* sequences generated by miss assembly of the Illumina reads. Therefore, the data regarding these CDS must be carefully interpreted and further validated.

### Putative non-coding RNA differentially expressed

In addition to the 559 CDS differentially expressed, our DEG analysis revealed the presence of 12 sequences up-regulated in females and five up-regulated in male mosquitoes currently annotated in *C. quinquefasciatus* genome as putative ncRNA (Table 1). The function of ncRNA in mosquitos’ physiology can be considered a recent area of study that is undergoing a rapid expansion. The advances of next-generation sequencing technologies in the last decades allowed the identification of thousands of putative ncRNA in different mosquito species, including *An. gambiae* [79], *Ae. aegypti* [80] and *C. quinquefasciatus* [81]. Though much of their function remains largely elusive, it is thought that they have regulatory roles in several biological processes such as transcriptional and post-transcriptional gene regulation, regulation of genomic stability and epigenic regulation [82–84].

The potential role of ncRNA during virus transmission is also under investigation. MicroRNA, 18–24 nucleotides ncRNA, were identified in *Aedes* spp. saliva and found to modulate viral replication in mosquito and mammalian cell cultures [85]. Likewise, microRNAs were also observed in the saliva and salivary glands of *An. coluzzii*, suggesting a role in the vector-host interface [86]. It is noteworthy that the ncRNA reported here are classified as long ncRNA since they are longer than 200 nucleotides. However, due to their abundance in female salivary glands (Supplementary file 1), we cannot discard their possible involvement in blood acquisition and/or pathogen transmission.

## Conclusion

In 2004 a Sanger-based sialome study of adult female *C. quinquefasciatus* was published and reported 503 protein sequences in which 284 were classified as putative secreted proteins [14]. In this work using an Illumina-based approach we reported 7,539 protein sequences from which 898 were classified as secreted proteins, providing a higher resolution of *C. quinquefasciatus* salivary gland contents. The differential expression analysis between males and females showed here allowed the identification of female-enriched putative secreted proteins. The present findings represent a comprehensive reference for future studies focused on the characterization of mosquito salivary proteins assisting in the identification and characterization of pharmacologically active proteins, proteins that can affect viral transmission and potential biomarkers of mosquito exposure.

## Electronic supplementary material

Below is the link to the electronic supplementary material.

**Additional file 1.**

## Data Availability

The transcriptome data was deposited at the National Center for Biotechnology Information (NCBI) under Bioproject PRJNA892574 and Biosample accession SAMN31387522. The raw reads were deposited at the Short Reads Archive of the NCBI under accessions SRR22053549 - SRR22053554. This Transcriptome Shotgun Assembly project has been deposited at DDBJ/EMBL/GenBank under the accession GKDO00000000. The version described in this paper is the first version, GKDO01000000.
