# Supplementary material for: A deeper insight into the sialome of male and female Culex quinquefasciatus mosquitoes
Source: BMC Genomics. 2023 Mar 20;24:135. doi: 10.1186/s12864-023-09236-1 (PMC10027276; doi:10.1186/s12864-023-09236-1)
Supplement: Supplementary file 1 — Additional file 1. [file 12864_2023_9236_MOESM1_ESM.docx]

**Supplementary Figures**

**
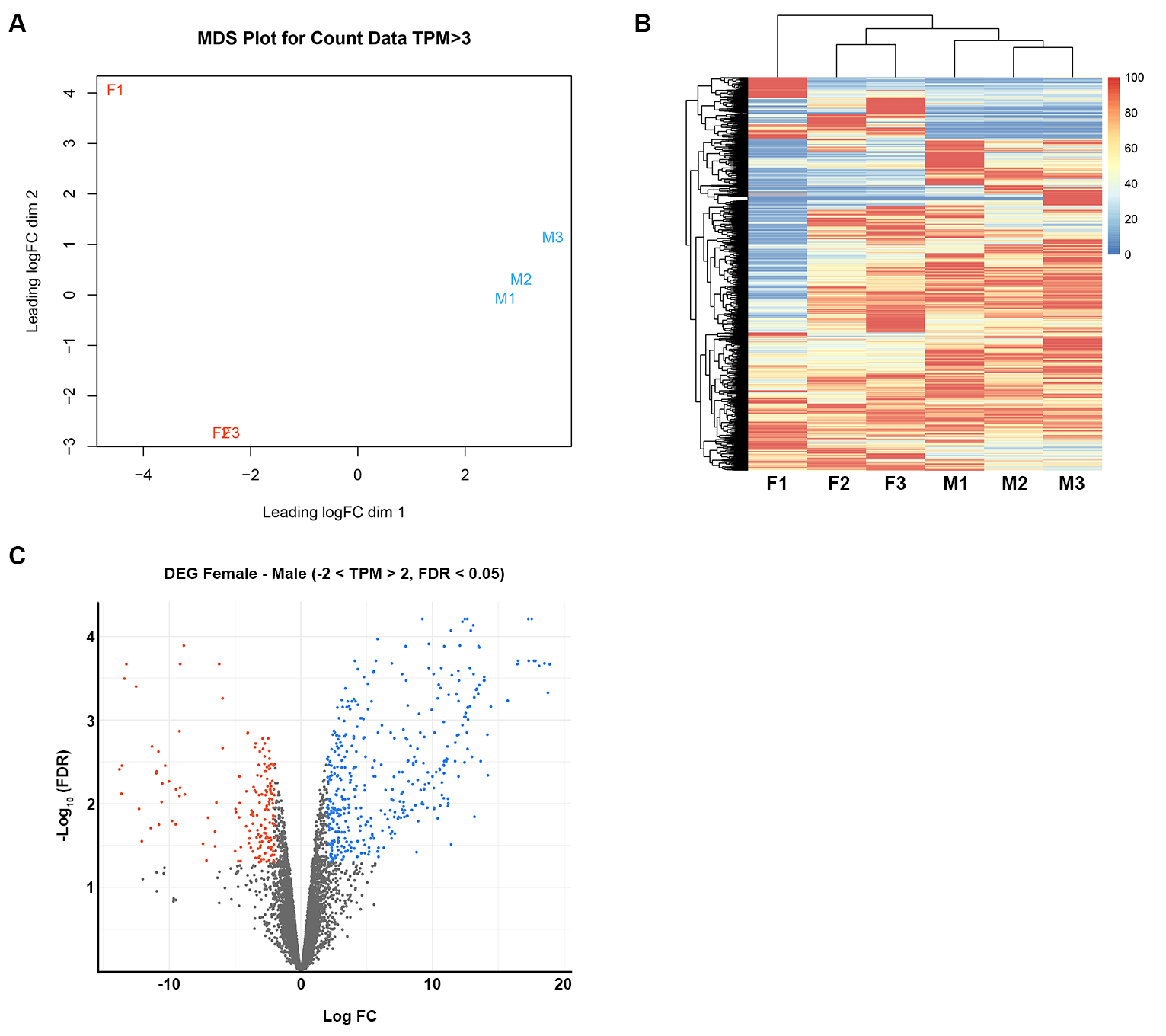
**

**Supplementary figure 1: (A):** Multidimensional plot based on the CDS identified in the salivary glands of females (F) and males (M) *C. quinquefasciatus* mosquitoes. **(B):** Heat map plot of the normalized TPM of each CDS identified in females (F) and males (M) *C. quinquefasciatus* mosquitoes. **(C):** Volcano plot of the CDS differentially expressed between female and male mosquitoes. The female down-regulated transcripts are shown in red while the up-regulated ones are represented in blue.

**
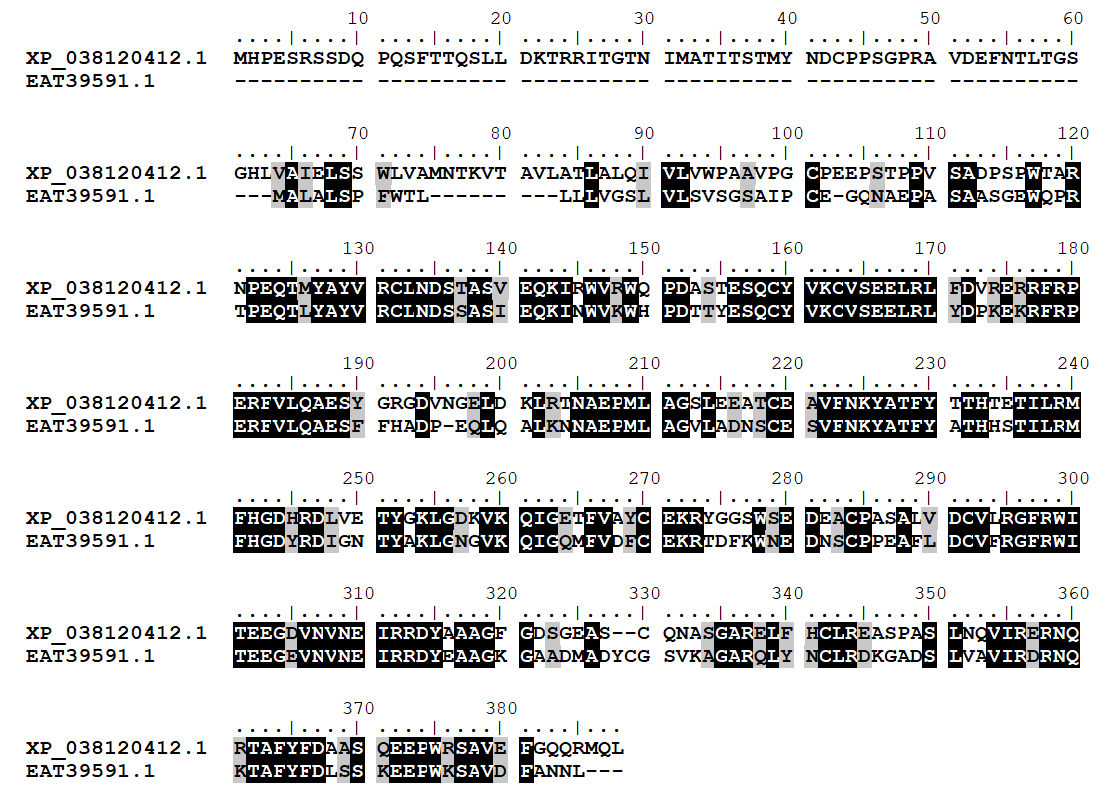
**

**Supplementary figure 2:** Amino acid alignment of the D7 proteins from *C. quinquefasciatus* (XP_038120412.1) and the juvenile hormone binding D7 from *Ae. aegypti* (EAT39591.1). Identical and similar residues are boxed.


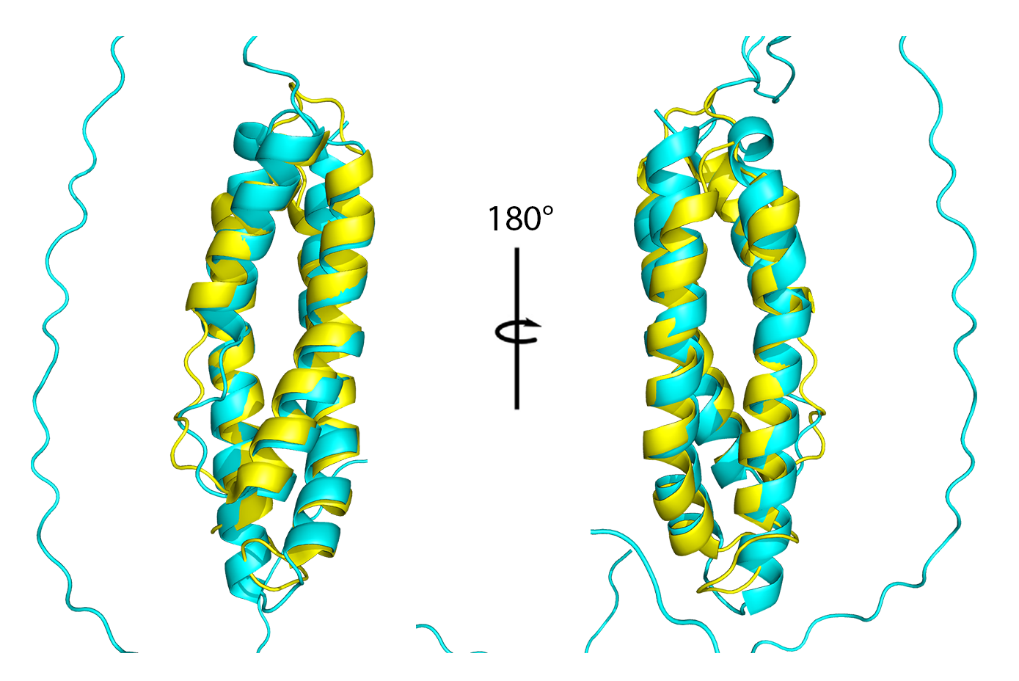


**Supplementary figure 3:** Structural superposition of albicin (yellow, PDB: 6XKE), the salivary complement inhibitor from *An. albimanus*, with the C-terminal region of aegyptin (cyan), the platelet aggregation inhibitor from *Ae. aegypti*. Aegyptin structure was modeled using AlphaFold2.


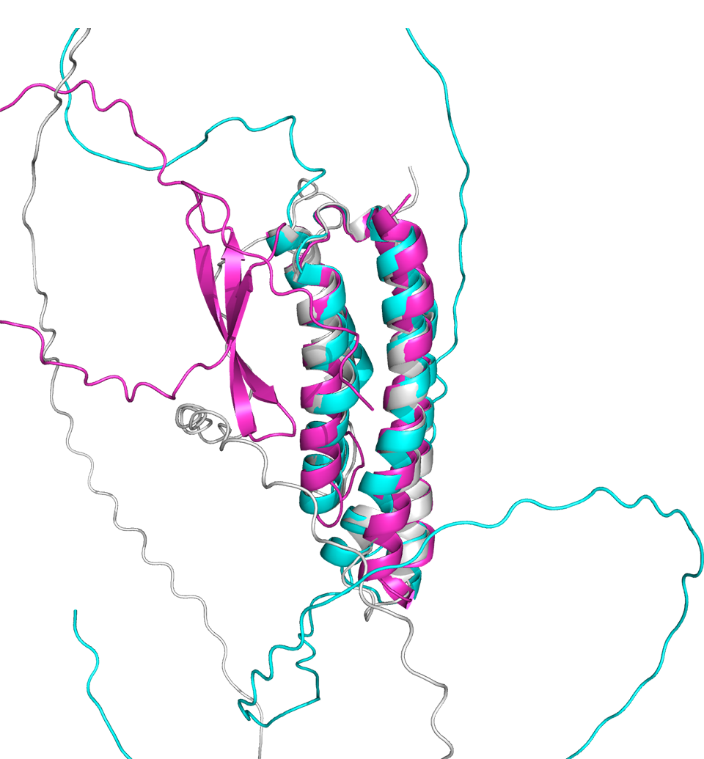


**Supplementary figure 4:** Superposition of XM_001845231 from *C. quinquefasciatus* (pink), aegyptin (cyan) from *Ae. aegypti* and AAPI from *An. stephensi* (gray). The three structures were modeled using AlphaFold2.


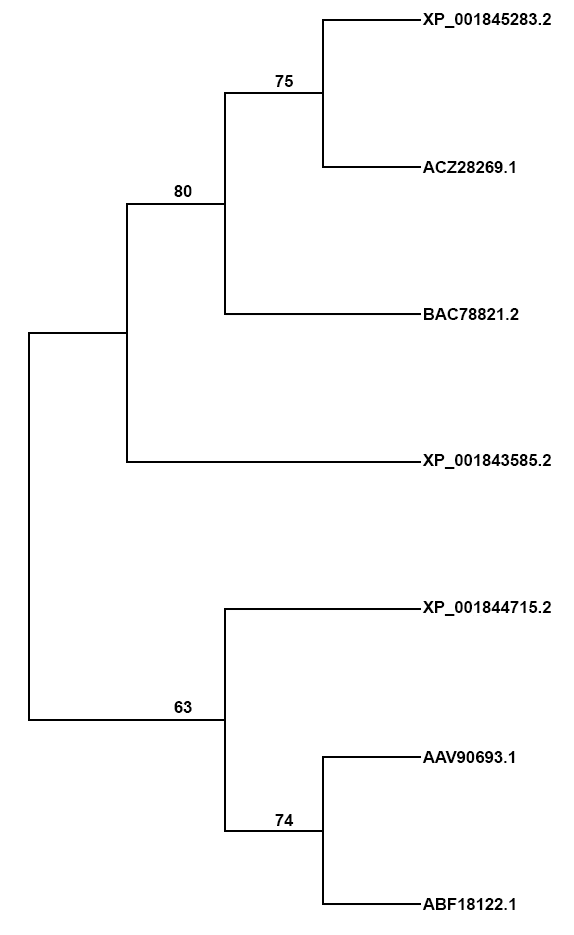


**Supplementary figure 5:** Phylogenetic tree of the 30 kDa salivary allergens from Diptera vectors. The numbers at the base of branches indicates the concordance between 500 bootstraps replicates. The three was constructed using the Maximum likelihood method with the Jones-Taylor-Thornton (JTT) model using MEGA11.

**
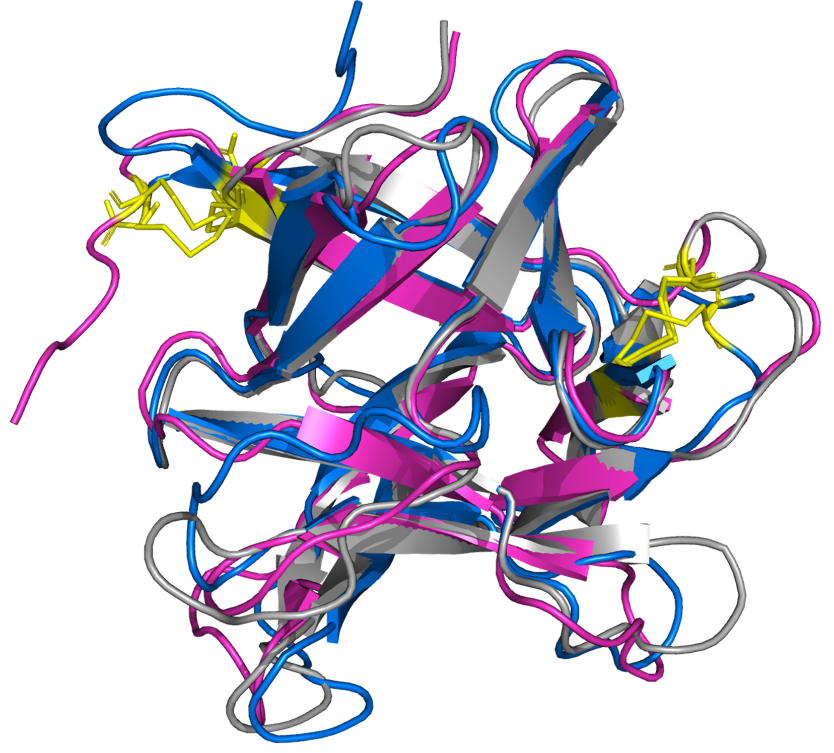
**

**Supplementary figure 6:** Superposition of putative salivary proteins containing the β-trefoil domain from *C. quinquefasciatus*. The crystal structure of CqDVP-2 (PDB: 7KC8) is shown in pink, while the alphafold model of XP_038107904.1 (blue) classified as a WRP protein and XP_038112911.1 (grey) from the 15.3 kDa protein family. Cysteine residues are represented as yellow sticks.

**Supplementary Table 1:** Relative quantification of putative CDS coding for long or short D7 proteins

| CDS | Average TPM in Female | Average TPM in Male | Protein family |
| --- | --- | --- | --- |
| XM_038258283.1 | 64497.91 | 6.09 | D7 LONG |
| XM_001865378.2 | 49918.57 | 2.85 | D7 LONG |
| XM_038251030.1 | 11581.13 | 175.82 | D7 LONG |
| XM_038255328.1 | 6391.33 | 61.74 | D7 LONG |
| XM_038255191.1 | 2248.46 | 0.01 | D7 LONG |
| XM_038251031.1 | 31.94 | 0.28 | D7 LONG |
| Cq-contig_13280 | 11.82 | 5.51 | D7 LONG |
| XM_038264484.1 | 70.82 | 27.55 | D7 LONG |
| XM_001865373.2 | 3780.23 | 0.49 | D7 SHORT |
| XM_038251032.1 | 3609.12 | 0.32 | D7 SHORT |
| XM_038256440.1 | 1134.02 | 0.30 | D7 SHORT |
| Cq-contig_16889 | 1052.61 | 0.13 | D7 SHORT |
| Cq-contig_990 | 40.21 | 0.04 | D7 SHORT |

**Supplementary files 1 and 2:** The hyperlinked excel spreadsheet and the associated files can be downloaded as a single zip file fromhttps://proj-bip-prod-publicread.s3.amazonaws.com/transcriptome/C_quinquefasciatus_sialome_2022/CQ_SUPPLEMENTARY_FILES.zip
